# Phytochemical Evaluation and Antioxidant-Antimicrobial Potential of *Lilium* spp. Bulbs: Therapeutic and Dermatocosmetic Applications

**DOI:** 10.3390/plants14131917

**Published:** 2025-06-22

**Authors:** Simona Lupșor, Gabriela Stanciu, Radu Emilian Cristache, Emilia Pănuș, Cristiana Radulescu, Radu Lucian Olteanu, Claudia Lavinia Buruleanu, Raluca Maria Stirbescu

**Affiliations:** 1 Department of Chemistry and Chemical Engineering, Faculty of Applied Sciences and Engineering, Ovidius University of Constanta, 900527 Constanta, Romania; sgutaga@univ-ovidius.ro; 2 Faculty of Medicine, Utrecht University, 3584 CS Utrecht, The Netherlands; r.e.cristache@students.uu.nl; 3 Constanța Public Health Directorate, 89 Nicolae Iorga Street, 900587 Constanta, Romania; temilia2@yahoo.com; 4 Faculty of Sciences and Arts, Valahia University of Targoviste, 130004 Targoviste, Romania; radu.olteanu@valahia.ro; 5 Doctoral School Chemical Engineering and Biotechnology, National University of Science and Technology Politehnica of Bucharest, 060042 Bucharest, Romania; 6 Academy of Romanian Scientists, 050044 Bucharest, Romania; 7 Faculty of Environmental Engineering and Food Science, Valahia University of Targoviste, 13 Sinaia Alley, 130004 Targoviste, Romania; lavinia.buruleanu@valahia.ro; 8 Institute of Multidisciplinary Research for Science and Technology, Valahia University of Targoviste, 130004 Targoviste, Romania; stirbescu.raluca@icstm.ro

**Keywords:** *Lilium* spp., maceration, antioxidant activity, phenolic compounds, flavonoids, condensed tannins, antimicrobial activity, FTIR spectroscopy, skin health, phytocosmetics

## Abstract

*Lilium* spp. bulbs are traditionally valued for their medicinal properties, yet their phytochemical profile and biomedical potential remain underexplored. This study aims to assess the antioxidant, antimicrobial, and dermatocosmetic potential of ethanolic macerates from five *Lilium* spp. cultivars. Bulb macerates were obtained using 70% and 96% ethanol and evaluated for total phenolic content (TPC), total flavonoid content (TFC), condensed tannins (CTC), mineral composition, and antioxidant activity (DPPH assay). Spectroscopic (FTIR) and antimicrobial analyses were also performed. Macerates from *Lilium* “Dark Secret” (LD-70) and *Lilium asiaticum* “White” (LA-70) exhibited the highest levels of TPC (225 and 162.5 mg GAE/100 g f.w.), TFC (26.12 and 21.75 mg QE/100 g f.w.), and antioxidant activity (81.5 and 58.75 mg GAE/100 g f.w.). FTIR confirmed the phenolic composition, while mineral analysis revealed a high potassium content and negligible toxic metals. Selective antimicrobial activity was observed against *Escherichia coli*, *Pseudomonas aeruginosa*, and *Candida albicans*, particularly for LD-70 and LA-70 macerates. Based on these findings, stable hydrogel formulations incorporating LD-70 and LA-70 were developed, showing favorable pH, rheology, and sustained antioxidant activity over 60 days. These findings support the integration of *Lilium*-derived macerates into dermatocosmetic formulations targeting skin protection and microbial defense.

## 1. Introduction

The *Lilium* genus, a member of the *Liliaceae* family, consists of more than 100 species distributed across the Northern Hemisphere temperate regions, including East Asia, Europe, and North America [1]. While *Lilium* species are primarily cultivated for ornamental purposes, they also hold significant medicinal and nutritional value [2,3]. In Traditional Chinese Medicine (TCM), *Lilium* bulbs, known as “Bai-he”, have been used for centuries to treat ailments such as chronic cough, hemoptysis, anxiety, insomnia, and palpitations [4,5]. European and Indian medicinal systems have also documented their use as galactagogues, diuretics, and treatments for inflammatory and skin-related conditions [1,4,5].

Although several *Lilium* species have documented therapeutic and cosmetic uses, many commercially available cultivars remain insufficiently studied regarding their antioxidant and antimicrobial potential. Addressing this knowledge gap is essential for identifying new natural ingredients with enhanced efficacy for pharmaceutical, nutraceutical, and cosmetic products [1,2,3].

Phytochemical investigations have revealed that *Lilium* spp. bulbs contain significant amounts of bioactive compounds, including phenolic acids, flavonoids, steroidal saponins, alkaloids, and polysaccharides [6]. In particular, phenolic compounds and flavonoids play a crucial role in scavenging free radicals, reducing oxidative stress, and protecting cellular integrity [7,8,9]. There is a strong correlation between total phenolic content (TPC), total flavonoid content (TFC), and antioxidant activity, reinforcing the importance of optimizing extraction techniques to maximize bioavailability [9,10]. Beyond their antioxidant potential, *Lilium* spp. macerates have demonstrated notable antimicrobial properties, with various species exhibiting activity against Gram-positive and Gram-negative bacterial strains, as well as fungal pathogens [10,11,12,13,14]. This antimicrobial efficacy is largely attributed to flavonoids, phenolic acids, and steroidal saponins, which have been shown to disrupt microbial cell walls and inhibit pathogen proliferation [7]. Additionally, the mineral composition of *Lilium* bulbs includes potassium, calcium, magnesium, and sodium, which contribute to their nutritional and therapeutic potential [6].

Studies on different *Lilium* species have highlighted their diverse bioactivities, ranging from anti-inflammatory, anti-tumor, hypoglycemic, antidepressant, hepatoprotective, and neuroprotective effects [4]. For instance, *Lilium candidum* L. has been reported to exhibit anti-diabetic effects through its modulation of pro-inflammatory cytokines and glucose uptake [4,5]. Similarly, macerates from *Lilium pumilum* and *Lilium regale* have demonstrated high levels of phenolic compounds and potent antioxidant activity, supporting their potential applications in nutraceutical and cosmetic formulations [13].

Despite the documented medicinal value of *Lilium* spp., most studies have focused on a limited number of species, leaving many ornamental and commercially cultivated cultivars underexplored in terms of their phytochemical composition and biomedical relevance. In particular, there is a lack of integrated studies that evaluate the antioxidant, antimicrobial, and mineral profiles of *Lilium* bulb macerates in parallel with their applicability in topical formulations. Given the increasing demand for natural, multifunctional ingredients in dermocosmetics and skincare, identifying plant-based actives with both protective and therapeutic properties is a timely research priority.

This study addresses this gap by systematically analyzing the chemical and biological profiles of ethanol macerates from five *Lilium* cultivars, including varieties not previously characterized. Furthermore, the study explores their formulation into hydrogel-based dermatocosmetic products, highlighting their potential as natural bioactive agents for skin protection and microbial control. Specifically, the influence of ethanol concentration (70% vs. 96%) on the extraction efficiency of bioactive compounds was investigated, assessing correlations between total phenolic content (TPC), total flavonoid content (TFC), condensed tannin content (CTC), and antioxidant activity using well-established methods such as the Folin–Ciocâlteu assay, DPPH radical scavenging assay, and vanillin–HCl assay. Additionally, Fourier-transform infrared (FTIR) spectroscopy was performed to characterize the functional groups present in the macerates, confirming information about their chemical composition. In addition to assessing safety through the detection of potentially toxic metals, the mineral profile was analyzed to evaluate the nutritional and functional relevance of essential elements, which may contribute to the biological activity and dermatological applicability of the macerates. In addition, antimicrobial activities were investigated against selected Gram-positive, Gram-negative, and fungal strains to evaluate their applicability as natural preservatives and active ingredients in dermatological and cosmetic products. Based on current scientific evidence, this study is among the few that investigates unexplored *Lilium* bulbs varieties. It provides new insights into their bioactive potential and expands current knowledge regarding their applicability in pharmaceutical and cosmetic formulations.

## 2. Results

### 2.1. Total Phenolic Content (TPC)

**Figure 1:**
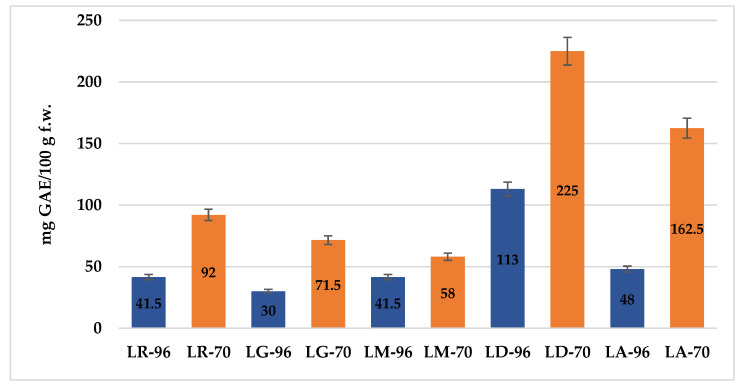
Total phenolic content (TPC) in *Lilium* spp. bulb macerates extracted with 70% and 96% ethanol. Results are expressed as mg gallic acid equivalents (GAE) per 100 g fresh weight. The macerates obtained with 70% ethanol are represented by orange bars, while those extracted with 96% ethanol are depicted as blue bars for each *Lilium* species. Values represent mean ± SD of triplicate measurements.

The results of the TPC determination, expressed in mg of gallic acid equivalents per 100 g of fresh weight (mg GAE/100 g f.w.), are presented in Figure 1. While the use of ethanol (70% vs. 96%) is well established, this study provides original quantitative data on the total phenolic content (TPC) of *Lilium* spp. bulb macerates. Given the known antioxidant and skin-protective properties of phenolics, these findings are particularly relevant for evaluating the potential dermatological and cosmetic applications of *Lilium* spp. macerates.

For clarity and conciseness in the presentation of results, the macerates obtained by maceration in 96% or 70% ethanol from the bulbs of the analyzed *Lilium* species will be referred to using the following abbreviations: LR-96 and LR-70 (*Lilium robina*), LG-96 and LG-70 (*Lilium* “Sunset boulevard”), LA-96 and LA-70 (*Lilium asiaticum* “White”), LM-96 and LM-70 (*Lilium candidum* L.—Madonna Lily), and LD-96 and LD-70 (*Lilium* “Dark Secret”).

Overall, extraction with 70% ethanol yielded higher TPC values across most species. Among the tested samples, LD-70 exhibited the highest TPC (225 mg GAE/100 g f.w.), followed by LA-70 with 162.5 mg GAE/100 g f.w., suggesting these bulbs are the richest in antioxidant compounds. In contrast, LG-96 showed the lowest TPC (30 mg GAE/100 g f.w.), indicating either a lower phenolic profile or reduced solvent efficiency at higher ethanol concentration.

### 2.2. Antioxidant Activity (AA)

**Figure 2:**
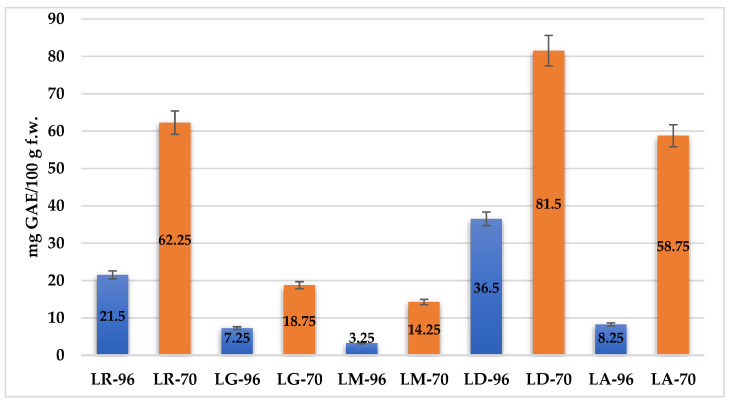
Comparison of radical scavenging activity (DPPH assay) of *Lilium* bulb macerates obtained using 70% ethanol (orange) and 96% ethanol (blue). Values are reported as mg gallic acid equivalents (GAE) per 100 g fresh weight. Error bars indicate the standard deviation.

Figure 2 presents the antioxidant activity of *Lilium* spp. bulb macerates, expressed as mg gallic acid equivalents per 100 g fresh weight (mg GAE/100 g f.w.), as determined by the DPPH radical scavenging assay.

The results reveal significant variability in antioxidant activity among species, as well as between solvent concentrations. The highest antioxidant activity was recorded for LD-70 (81.5 mg GAE/100 g f.w.), followed by LR-70 (62.1 mg GAE/100 g f.w.) and LA-70 (58.75 mg GAE/100 g f.w.). Conversely, the lowest AA was observed in LM-96, with a value of 3.25 mg GAE/100 g f.w.

### 2.3. Total Flavonoid Content (TFC)

**Figure 3:**
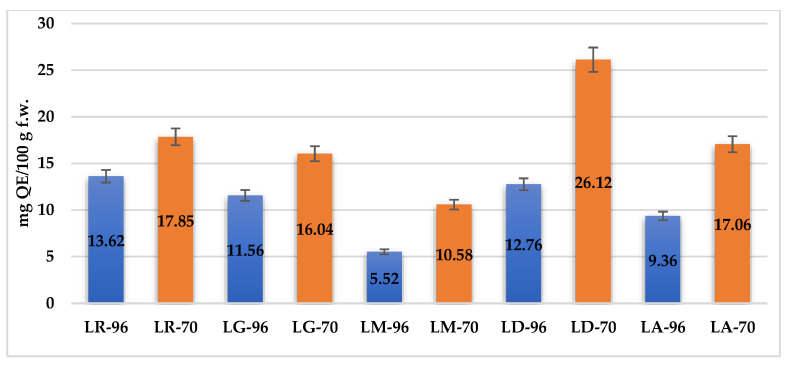
Total flavonoid content (TFC) of *Lilium* spp. bulb macerates, expressed as mg of quercetin equivalents per 100 g fresh weight (mg QE/100 g f.w.). Orange bars represent macerates obtained with 70% ethanol, while blue bars correspond to those extracted with 96% ethanol. Error bars indicate the standard deviation.

The TFC of *Lilium* spp. bulb macerates, expressed as milligrams of quercetin equivalents per 100 g of fresh weight (mg QE/100 g f.w.), exhibited significant variation across species and solvent concentrations, as depicted in Figure 3.

The highest values were observed in LD-70 (26.12 mg QE/100 g f.w.) and LA-70 (21.75 mg QE/100 g f.w.), followed by LR-70 (17.5 mg QE/100 g f.w.) and LM-70 (15.0 mg QE/100 g f.w.). In contrast, LG-96 exhibited the lowest TFC, with 4.37 mg QE/100 g f.w. Overall, macerates obtained from LD and LA cultivars demonstrated the most favorable flavonoid profiles, reinforcing their potential as sources of antioxidant compounds.

### 2.4. The Condensed Tannins Content (CTC)

The CTC of *Lilium* spp. bulb macerates was evaluated using 70% ethanol as the extraction solvent. This solvent concentration was chosen based on prior studies indicating that moderate ethanol levels (40–70%) significantly enhance the extraction efficiency of polyphenolic compounds, including condensed tannins, in comparison with absolute ethanol (96%) [15,16,17]. Furthermore, given the naturally low tannin content in *Lilium* bulbs, the use of 70% ethanol was considered optimal for maximizing the extraction yield and improving the probability of successful quantification of condensed tannins.

The CTC of the *Lilium* spp. bulb macerates were analyzed, with results presented in Table 1. The results show that only LM-70 and LD-70 contained quantifiable levels of condensed tannins, while the other macerates had non-detectable (n.a.) values.

Among the analyzed *Lilium* spp. macerates, the highest CTC values were recorded for LD-70, followed by LM-70.

These values, however, remain significantly lower than those reported in the literature for other tannin-rich plant species, suggesting that *Lilium* bulbs are a poor natural source of condensed tannins. These findings reinforce the importance of solvent polarity in optimizing tannin extraction. Previous studies [15,16] have reported that alcohol concentrations outperform ethanol (96%) in extracting condensed tannins, which supports our findings.

The limited presence of tannins in these macerates may be attributed to species-specific metabolic differences and the inherently low tannin biosynthesis in *Lilium* bulb. Further studies should explore alternative extraction methods or additional species within the *Lilium* genus to better understand the distribution of condensed tannins in this plant family, only for LM-70 and LD-70.

According to the *t*-test, all the samples were different in terms of total polyphenolic content (TPC), total flavonoid content (TFC), and antioxidant activity (AA), respectively (*p* < 0.05), regardless of the solvent used for extraction.

**Figure 4:**
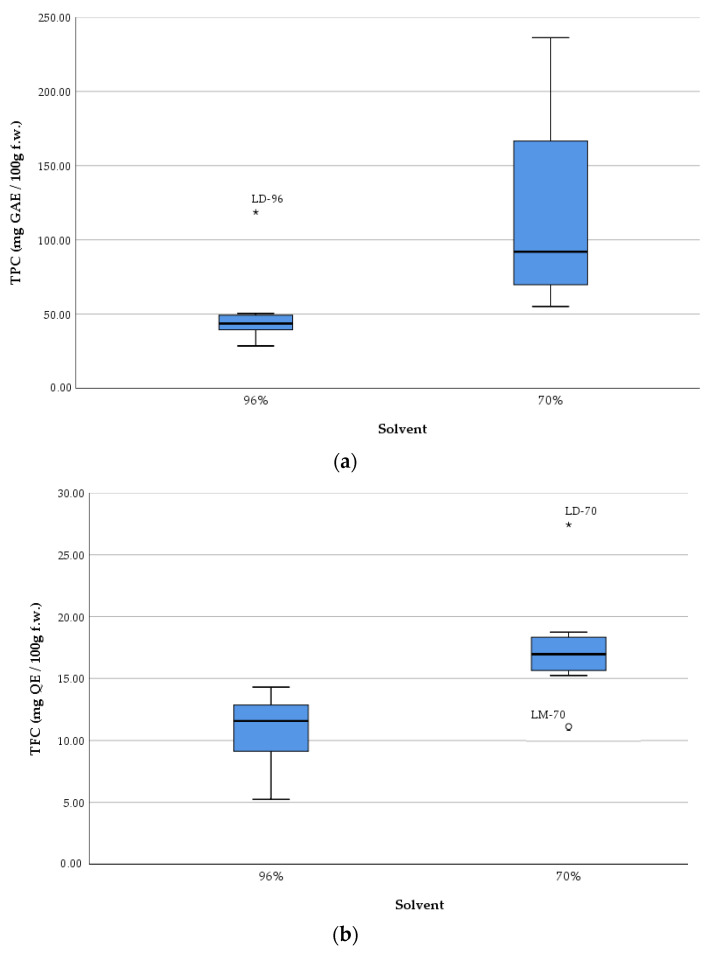

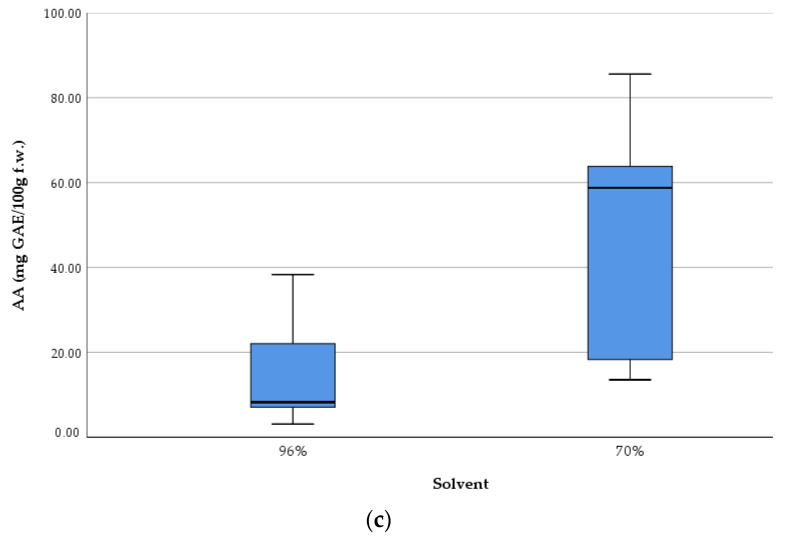
Comparative boxplots of total polyphenolic content (TPC) (**a**), total flavonoid content (TFC) (**b**), and antioxidant activity (AA) (**c**) of *Lilium* spp. macerates. In (**a**), the asterisk (*) related to sample LD-96 shows the TPC value of this sample, the highest compared to all values in the data set. In (**b**), the asterisk (*) related to sample LD -70 shows the TFC value of this sample, the highest compared to all values in the data set. In Figure (**b**), the circle (◦) related to sample LM -70 shows the TFC value of this sample, the smallest compared to all values in the data set.

The boxplots (Figure 4) show the measures of central tendency and dispersion of data, as well as detect extreme values (outliers). The graphical representation underlines, once again, the efficiency of the solvent with 70% ethanol compared to that with 96% ethanol concerning the extraction of TPC and TFC from samples, respectively increasing their antioxidant activity.

The boxplots show an overall picture of the median values of each parameter and on the outliers (i.e., LD-96 sample in the case of TPC, respectively LD-70 and LM-70 in the case of TFC).

Strong and statistically significant positive correlations were observed among TPC, TFC, and AA, indicating a close interdependence between these phytochemical parameters (Table 2).

A strong and statistically significant positive correlation was observed between AA and both TPC (*r* = 0.907, *p* < 0.01) and TFC (*r* = 0.895, *p* < 0.01), indicating that these compounds play a major role in the antioxidant potential of *Lilium* spp. macerates. Furthermore, a strong correlation was also found between TPC and TFC (*r* = 0.839, *p* < 0.01), suggesting that flavonoids constitute a considerable proportion of the total polyphenols.

These results reinforce the well-established contribution of polyphenolic compounds, particularly flavonoids, to antioxidant activity.

**Figure 5:**
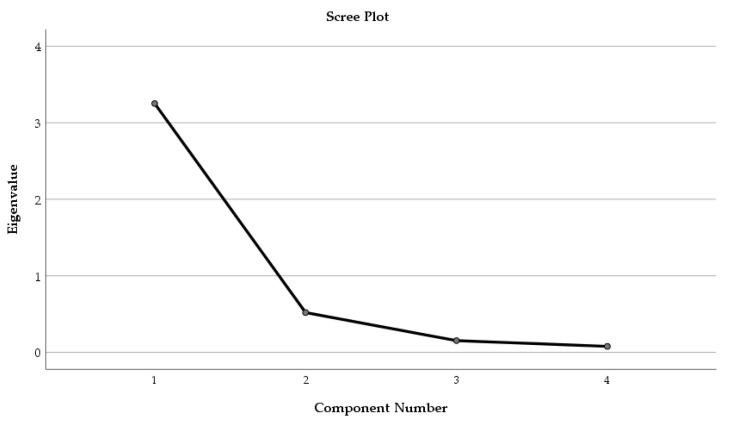
Screen Plot showing the extraction of components during Principal Component Analysis.

Factor Analysis was applied to the phytochemical parameters (TPC, TFC, and AA) and the solvent used for extraction (Figure 5).

Only one component was extracted. The total variance explained by the four parameters (TPC, TFC, AA, and solvent, respectively) was 81.28%. The component matrix was structured, and the factor loading of each parameter was established as follows: TPC—0.928, TFC—0.945, AA—0.957, and solvent—0.762, respectively.

Hierarchical Cluster Analysis took into consideration all 10 samples and their associated content in TPC and TFC, respectively, and their antioxidant activity. Between-groups linkage method was applied, and the Squared Euclidean distance was selected for the interval.

**Figure 6:**
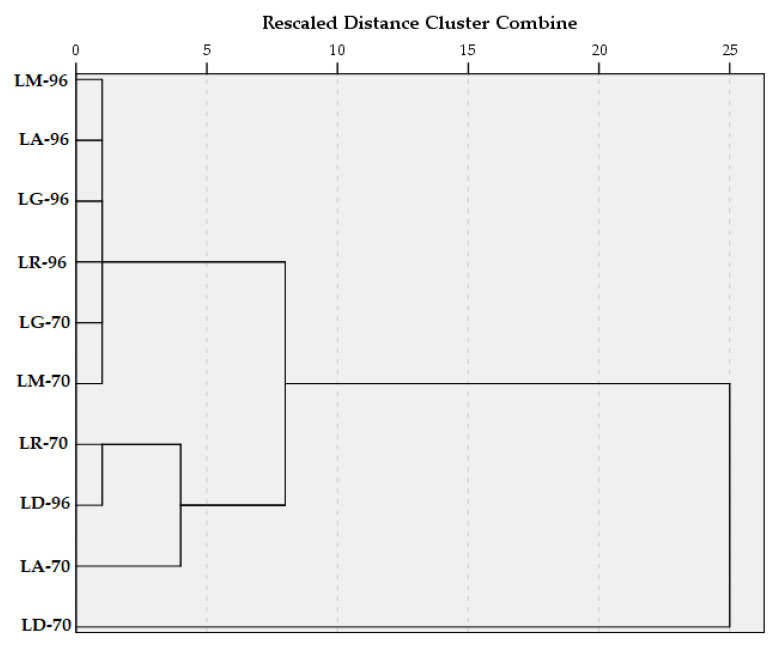
Dendrogram using Average Linkage (Between Groups).

The dendrogram (Figure 6) shows that in the first clustering stage, one group was structured from the samples with similar TPC, TFC, and AA values, namely LM-96, LA-96, LG-96, LR-96, LG-70, and LM-70. Another cluster contains only two samples (LR-70 and LD-96), and the sample LA-70 later joined it. In the third stage of clustering, all the samples, except LD-70, were grouped into one cluster. This was due to the remarkable amount of TPC and TFC compared with the other macerates.

These findings suggest that both the *Lilium* species and the solvent concentration significantly influence the phenolic profile and antioxidant activity of the macerates.

### 2.5. Metal Concentrations

**Figure 7:**
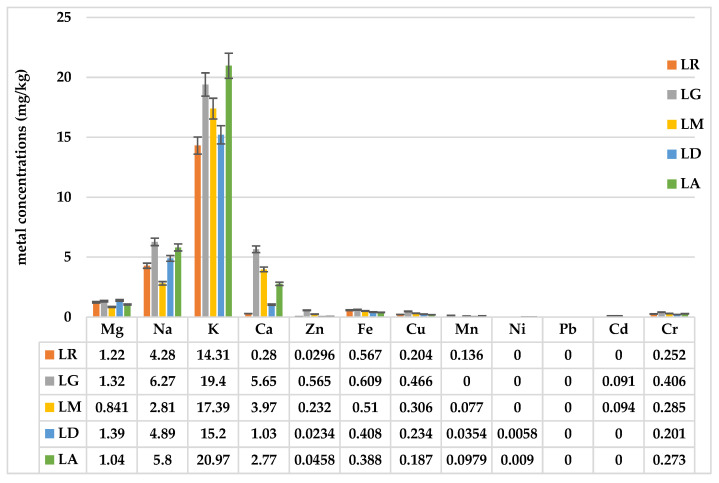
Mineral content of *Lilium* spp. bulbs samples. Bars represent the concentrations of key macrominerals—potassium (K), sodium (Na), calcium (Ca), magnesium (Mg)—as well as important micronutrients (e.g., iron (Fe), zinc (Zn), copper (Cu), manganese (Mn)) and any detected heavy metals, expressed in mg per kg of sample. Error bars indicate the standard deviation.

The analysis of the mineral content in *Lilium* spp. bulb macerates revealed notable differences in key elements among the samples studied (Figure 7). Variations in mineral concentrations, including potassium (K), sodium (Na), calcium (Ca), and magnesium (Mg), suggest species-specific differences in nutrient accumulation, which could influence their potential applications in pharmaceuticals, cosmetics, and nutrition.

Among the analyzed minerals, potassium (K) was the most abundant mineral, with the highest concentration observed in *Lilium asiaticum* “White” (LA) (20.97 mg/kg), followed by *Lilium* “Sunset boulevard” (LG) (19.4 mg/kg). Calcium (Ca) levels varied significantly, with *Lilium* “Sunset boulevard” (LG) showing the highest concentration (5.65 mg/kg), while *Lilium robina* (LR) contained the lowest (0.28 mg/kg). Magnesium (Mg) concentrations were relatively consistent across samples, ranging from 0.841 mg/kg in *Lilium candidum L.* (LM) to 1.39 mg/kg in *Lilium* “Dark secret” (LD) while sodium (Na) was highest in *Lilium* “Sunset boulevard” (LG) (6.27 mg/kg) and lowest in *Lilium candidum L.* (LM) (2.81 mg/kg).

Essential trace metals such as iron (Fe), zinc (Zn), copper (Cu), and manganese (Mn) were present in variable amounts, with iron (Fe) levels highest in LG (0.609 mg/kg), suggesting a potential role in enzymatic and metabolic functions [18].

The analysis of toxic metals confirmed that lead (Pb) and cadmium (Cd) were either absent or detected in negligible amounts, ensuring the safety of *Lilium* spp. bulbs for potential applications. Nickel (Ni) was detected at low levels in LA (0.009 mg/kg) and LD (0.0058 mg/kg), while chromium (Cr) was found at the highest levels in LG (0.406 mg/kg).

### 2.6. Fourier Transform Infrared (FTIR) Spectroscopy

**Figure 8:**
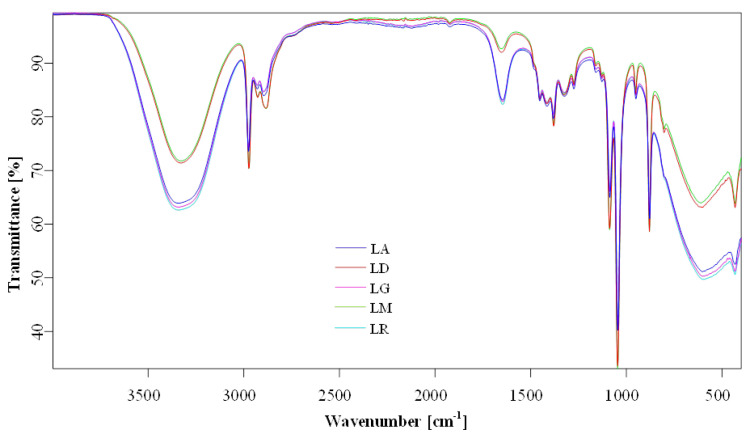
Overlapping FTIR spectra of *Lilium* spp. bulb macerates (blue—LA, red—LD, pink—LG, green—LM, and turquoise—LR). LA—*Lilium asiaticum* “White”; LD—*Lilium* “Dark Secret”; LG—*Lilium* “Sunset boulevard”; LM—*Lilium candidum* L. “Madonna Lily”; LR—*Lilium robina*.

The overlapping FTIR spectra of *Lilium* sp. macerates samples (i.e., LR-96, LR-70; LD-96, LD-70; LG-96, LG-70; LM-96, LM-70; and LA-96, LA-70) are presented in Figure 8. The chemical characterization in terms of group assignments (Table 3) was similar to data reported by Thi et al. (2017) [19] and Munafo Jr and Gianfagna (2015) [20]. The most prominent features were phenylpropanoid-associated peaks of *Lilium* spp. macerates, observed around 1647–1654, 1160, 878, and 950 cm^−1^, in the FTIR spectra (all vibrations are related to phenyl ring vibrations, Table 3). On the other hand, a strong and broad band at 3328–3341 cm^−1^ was attributed to the stretching vibration mode of O-H groups and a strong band at 1044 cm^−1^ for the stretching vibration mode of the same group. The wide-ranging band, as a week to medium peaks, from 2884 cm^−1^ *Lilium candidum* L. (LM) to 2897 cm^−1^ *Lilium* “Sunset boulevard” (LG), can be assigned to the bending vibration of O-H and C-O/C=O groups corresponding to the caffeic acid [19,21]. Weak bands at 1382 and 1274 cm^−1^ were assigned to C-O stretching vibration combined with phenyl ring and C-C in-phase (symmetric motion) ring stretching, respectively.

The spectra highlight the presence of phenolic and flavonoid functional groups. Peaks are compared for structural similarities across cultivars. Detailed peak assignments are listed in Table 3.

### 2.7. Antimicrobial Activity

The bacterial and fungal strains selected—*Staphylococcus aureus* ATCC 25923, *Escherichia coli* ATCC 25922, *Pseudomonas aeruginosa* ATCC 27853, and *Candida albicans* ATCC 10231—are clinically relevant pathogens frequently associated with skin, wound, and nosocomial infections, making them suitable models for assessing the antimicrobial potential of natural macerates [1,4,22]. The antimicrobial activity of *Lilium* macerates (LA-70, LD-70, LG-70, LM-70, and LR-70) was chosen for their proven superior ability to solubilize phenolic and flavonoid compounds with known antimicrobial properties. As summarized in Table 4, the results demonstrated strain-dependent antimicrobial responses influenced by the type of macerate and the volume applied.

The Kirby–Bauer disc diffusion method on Mueller–Hinton agar was employed following inoculation with a 0.5 McFarland suspension, and zones of inhibition were measured to determine sensitivity across sample volumes ranging from 5 to 30 µL.

The results revealed distinct responses among Gram-positive and Gram-negative bacteria, as well as the yeast, depending on both macerate type and volume. Macerates from LA-70 and LG-70 exhibited both antibacterial and antifungal activities. LG-70 showed antimicrobial effects across all tested volumes, while LA-70 and LD-70 were particularly effective against Gram-negative bacteria and *Candida albicans*. *Staphylococcus aureus* was inhibited only at higher macerate volumes (≥20 µL). Notably, LA-70 demonstrated greater antimicrobial efficacy than LD-70, highlighting its potential as a bioactive agent.

A comparative analysis of inhibition zones showed that *E. coli* was most sensitive to LA-70 and LD-70, with inhibition zones up to 19 mm at 30 µL. *S. aureus* showed moderate sensitivity to LG-70 (up to 18 mm), while *P. aeruginosa* was the least susceptible, despite responding to all macerates. *C. albicans* was particularly sensitive to LD-70 and LA-70, with inhibition zones reaching 22 mm, indicating strong antifungal potential.

Our findings align with the existing literature on the antimicrobial properties of *Lilium* species [22]. For instance, macerates from *Lilium candidum L.* (LM-70) have demonstrated antibacterial activity against pathogens such as *Escherichia coli*, *Pseudomonas aeruginosa*, and *Staphylococcus aureus*, with inhibition zones ranging from 5.33 mm to 18.88 mm, depending on the bacterial strain and macerate concentration.

A *t*-test was applied to macerates obtained by using 70% ethanol, taking into account their chemical parameters (TPC, TFC, AA, and CTC) and microbial parameters (diameter of inhibition zone of growth of the four pathogens previously mentioned), respectively. The samples were different in terms of their chemical parameters (*p* < 0.05) but not in terms of microbiological ones (*p* > 0.05), as they were determined in this study.

One-Way ANOVA also showed that there was no significant variation between groups (*p* > 0.05) if the antimicrobial properties of macerates are discussed. Because groups vary along more than one factor, two-factor ANOVA evaluated the influence of two independent variables (the tested organism and antioxidant activity) on a continuous dependent variable (the inhibition zone of growth). The analysis revealed that there are main effects both for the tested organism (Sig. = 0.000) and antioxidant activity (Sig. = 0.001), respectively, and, also, if the four micro-organisms are discussed, interaction between organism and antioxidant activity of the macerates (Sig. = 0.014). Thus, this interaction should be exploited in future research to select a suitable combination between the antioxidant activity of macerates and the targeted micro-organisms of interest.

Positive significant correlations were determined between TPC, TFC, AA, and CTC, respectively (Table 5). No correlations were established between the inhibition zone of growth and the chemical parameters.

Strong correlations (r > 0.75) were determined for TPC, TFC, and AA, respectively. Significant correlations were also established between CTC and TPC (r = 0.404) and CTC and TFC (r = 0.310), but both relationships are weak.

**Figure 9:**
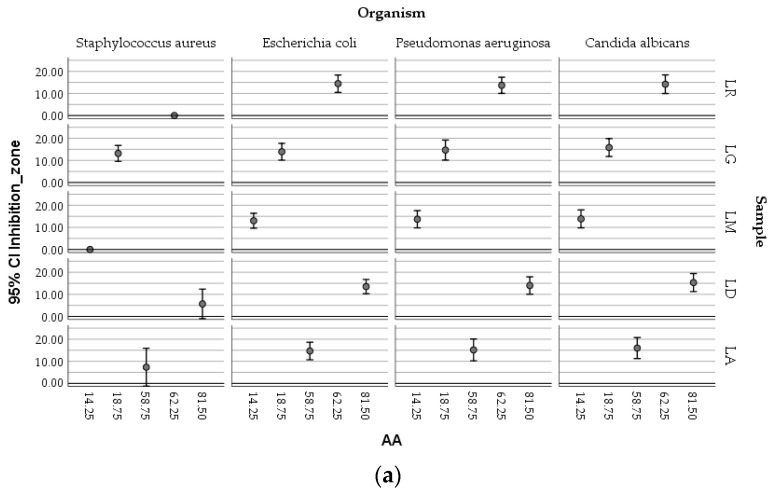

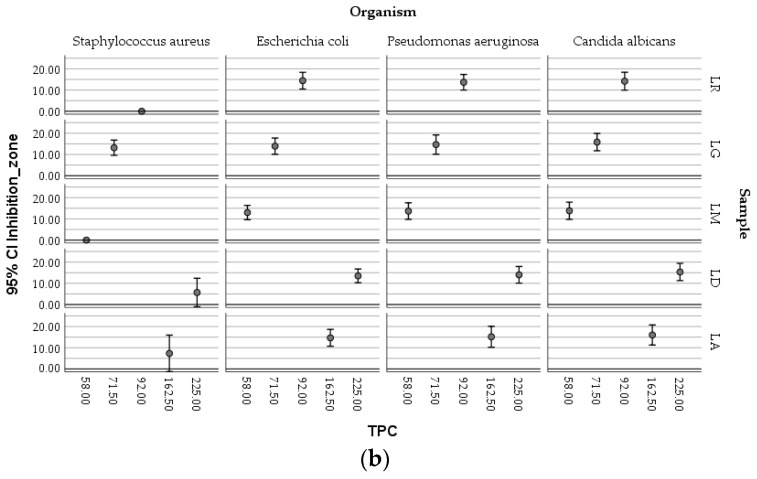
Boxplot matrices showing the relationships between the antimicrobial activity of *Lilium* macerates (expressed as inhibition zone), total phenolic content (TPC), and antioxidant activity (AA). Panel (**a**): Inhibition zone vs. antioxidant activity for each tested micro-organism (*S. aureus* ATCC 25923, *E. coli* ATCC 25922, *P. aeruginosa* ATCC 27853, and *C. albicans* ATCC 10231). Panel (**b**): Inhibition zone vs. TPC.

The boxplots (Figure 9) showed the complex relationships existing between the antimicrobial activity of ethanolic macerates (expressed as inhibition zone of organism growth), their antioxidant activity, and the concentration of TPC, respectively, depending on the tested micro-organisms and the *Lilium* sample.

Higher AA values determined in the case of LD-70 were obviously efficient against the growth of *Candida albicans* compared with *Staphylococcus aureus*. The LG-70 samples proved to inhibit moderate *S. aureus* ATCC 25922, the antioxidant activity of the corresponding macerates being, however, lower, meaning that other factors could also be responsible for the inhibitory effect on this bacterial strain. Similar inhibitory activities against *Escherichia coli* were determined in relation to different values of the antioxidant activity associated with different macerates.

The TPC values followed a similar distribution within the mapping of the parameters.

Considering the potential impact of the phytochemical compounds on the resulting antimicrobial activity of the samples, a Factor Analysis (FA) was performed to summarize their association. Principal Component Analysis (PCA) was selected as the extraction method.

The results of the Factor Analysis showed that PC1 and PC2 accounted for a cumulative data variance of 78.96%, PC1 explaining a high percentage of the total variance (56.78%) (Table 6).

**Figure 10:**
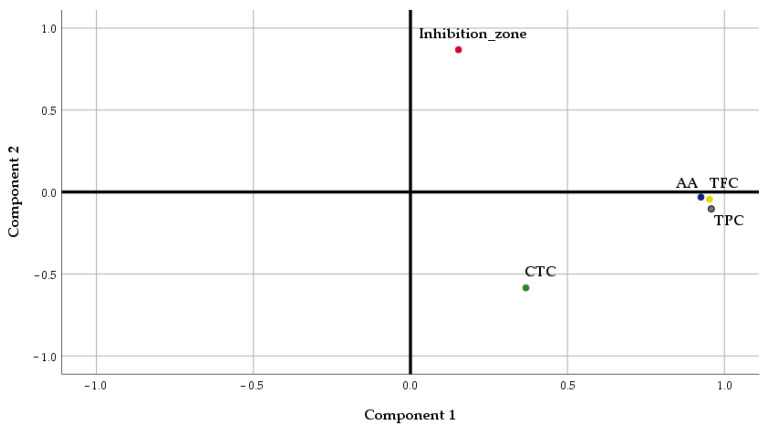
Principal Component Analysis of phytochemical compounds of macerates and their antimicrobial activity against the tested strains (Component Plot in Rotated Space). TPC—Total polyphenolic content (mg gallic acid/100 g of fresh weight); AA—antioxidant activity (mg gallic acid/100 g fresh weight); TFC—Total flavonoid content (mg quercetin/100 g fresh weight); CTC—Condensed tannins content (mg (+)-catechin eq./100 g of fresh weight).

TPC, TFC, and AA were located in the positive part of PC1, in agreement with the results of the previous analysis, underlying the strong relationships existing between antioxidant activity and the content of phenolics and flavonoids of *Lilium* spp. macerates (Figure 10). The total content of tannins was situated in the negative part of PC1, explaining the weak relationship of this parameter with TPC and TFC, as was determined through Pearson’s analysis. PC2 is formed only by the inhibition zone of micro-organisms’ growth.

The interpretation of PC1 and PC2 loadings suggests that high AA, TPC, and TFC values, respectively, and small CTC values contribute to higher antimicrobial activity expressed as the diameter of the inhibition zone.

**Figure 11:**
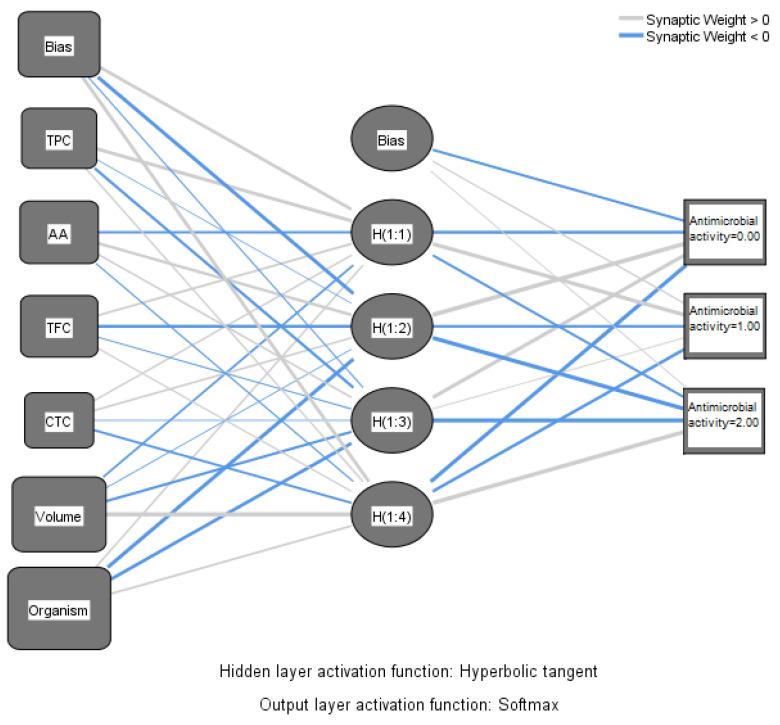
Neural network of data (Multilayer Perceptron). TPC—Total polyphenolic content (mg gallic acid/100 g of fresh weight); AA—antioxidant activity (mg gallic acid/100 g fresh weight); TFC—Total flavonoid content (mg quercetin/100 g fresh weight); CTC—Condensed tannins content (mg (+)-catechin eq./100 g of fresh weight).

Machine learning is preferable in some circumstances when the association between predictors and outcomes cannot be sufficiently explained through regression analysis. In this research, neural network analysis was applied with a view to correlating the antimicrobial activity of the alcoholic macerates (70% ethanol) expressed as inhibition zone of pathogens’ growth with the chemical parameters and also with the four tested pathogenic organisms (Figure 11).

The circles (nodes) containing the antioxidant activity (AA), total phenolics content (TPC), total flavonoid content (TFC), and total condensed tannins (CTC), respectively the type of micro-organism and the volumes of macerates tested, are linked through lines creating the connection between these ones and the output (resulting antimicrobial activity). The output was expressed as the diameter of the inhibition zone of growth, on a scale with three intervals as follows: none (0 mm); weak (6–12 mm), and strong (>12 mm). Thus, the output layer comprises three nodes, representing the intensity of the antimicrobial activity. The boxes called bias have the role of correcting systematic errors in the predictions.

Four nodes corresponding to four hidden layers were designed in SPSS. Activation from the hidden layers goes to antimicrobial activity weighted by the various data. The thick lines indicate that the weight diverges from “0” considerably. The weight exceeding “0” is indicated with blue lines.

Table 7 of the parameter estimates primarily specifies the biases and weights of the parameters involved in the analysis.

**Figure 12:**
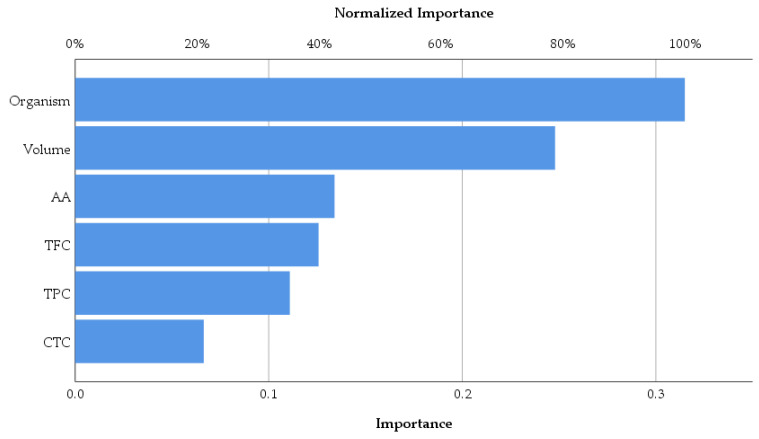
Normalized importance of the chemical parameters on the antimicrobial activity of macerates according to neural network analysis.

As was expected, the antimicrobial activity of macerates is dependent on the type of organism and also by the volume of macerate used in analysis. Overall, according to the data represented in Figure 12, the antioxidant activity of macerates affects the antimicrobial activity in the percentage of 42.5% within the group of the tested inputs. Also, TFC affects the same output more than TPC and CTC, respectively. Therefore, *Lilium* spp. alcoholic macerates with high antioxidant activity and total flavonoid content seemed to lead to increased antimicrobial activity against the four tested organisms. The neural network analysis revealed this way the importance of the total flavonoid content of macerates compared to the total phenolic content in terms of their antibacterial and antifungal activity.

### 2.8. Characteristics of Hydrogels Based on Lilium spp. Bulb macerates

*Lilium* “Dark Secret” (LD-70) and *Lilium asiaticum* “White” (LA-70) were selected as the lead candidates due to their exceptional total phenolic content, total flavonoids content, and antioxidant activity. The characteristics of the analyzed hydrogels are presented in Table 8.

The two hydrogels analyzed show homogeneity, indicating a uniform distribution of the active ingredients in the formulations. Stability is good as no phase separation, sedimentation, or visible changes in texture were observed over time, highlighting their robustness under standard storage conditions. In addition, their pH values are compatible with the physiological pH of the skin, making them suitable for long-term dermal application. In general, it is important for dermatocosmetic formulations to maintain a pH within the natural range of the skin (4.5–5.5) to avoid irritation, disruption of the skin barrier, or interference with the natural skin microbiota. Products that are too alkaline can remove natural oils, increase dryness, and damage the skin barrier, while products that are too acidic can cause irritation or sensitivity, especially in damaged or sensitive skin types [23,24]. In addition, the viscosity of the formulations showed minimal variation over time, indicating consistent rheological behavior and optimal spreading. As can be seen from Table 8, the organoleptic characteristics of the analyzed gels remain unchanged over time (after 30 and 60 days from preparation), and there are very small variations in terms of pH and viscosity.

**Figure 13:**
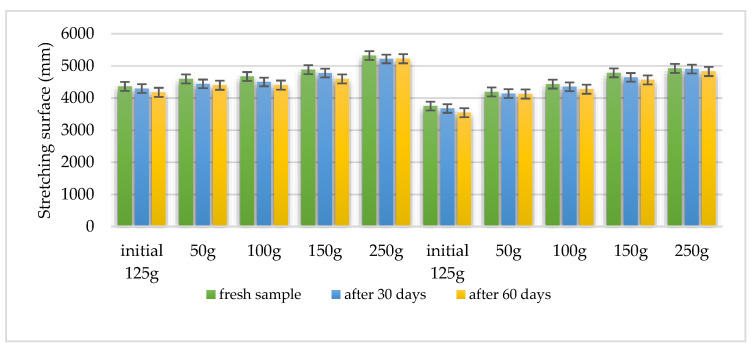
Spreadability of hydrogel HLD-70 (*Lilium* “Dark Secret”—ethanol 70% macerate).

**Figure 14:**
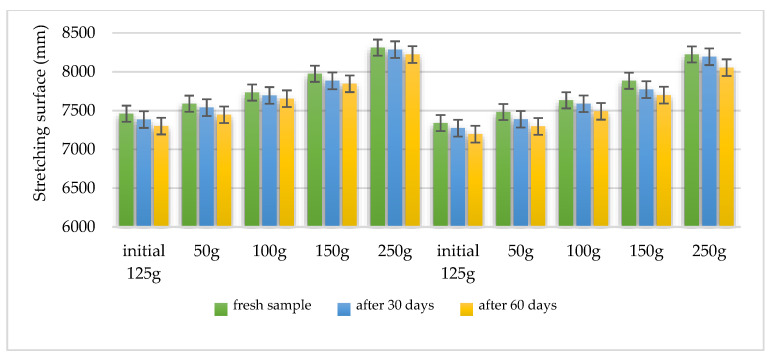
Spreadability of hydrogel HLA-70 (*Lilium asiaticum* “White”—ethanol 70% macerate).

Using the Ojeda Arboussa method for analysis [23], the graphs shown in Figure 13 and Figure 14 illustrate a high degree of spreadability for the HLD-70 (Hydrogel *Lilium* “Dark secret”) and HLA-70 (Hydrogel *Lilium asiaticum* “White”) gels. The two gels exhibit comparable spreadability. Furthermore, the spreadability parameters remained stable over time, indicating the consistency of the formulations and suitability for prolonged use. These findings suggest that the physical characteristics of the gels are well balanced to ensure effective application and user satisfaction.

Spreadability measured using the Ojeda Arboussa method. Values reflect average spread diameter (mm) on glass surface under constant weight.

Measurements reflect stable spreadability over time, determined by standard dermato-formulation methods.

**Figure 15:**
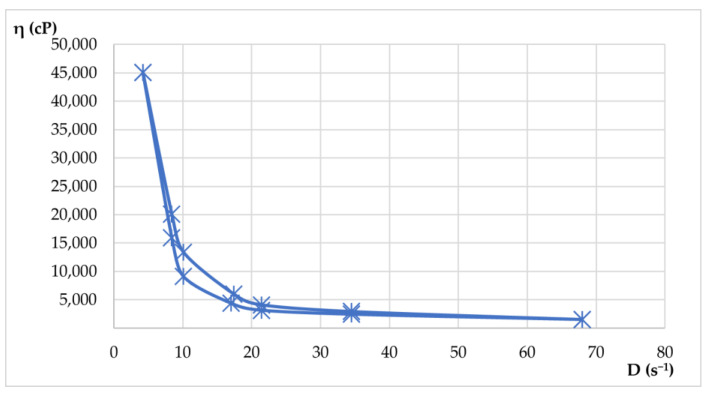
Flow curve for hydrogel HLD-70 (*Lilium* “Dark Secret”—ethanol 70%).

**Figure 16:**
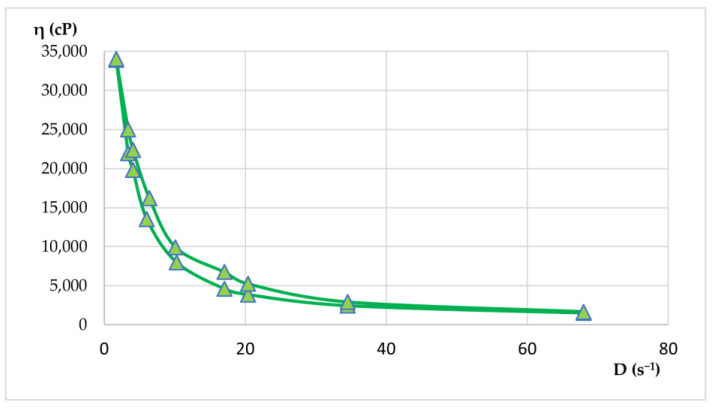
Flow curve for hydrogel HLA-70 (*Lilium asiaticum* “White”—ethanol 70%).

Rheological properties were evaluated by viscosity measurements at various rotational speeds, both up and down. From these measurements, shear rate and shear stress values were calculated. With the average values, rheograms and flow curves were generated to illustrate the flow behavior of the materials. Apparent viscosity readings (η, in cP) were taken in triplicate for each rotational speed (ω, in rpm) in both the increasing and decreasing phases. The results of the rheological determinations are presented in Table 9 and Table 10 and visualized in Figure 15 and Figure 16.

Both hydrogels exhibited favorable viscosity profiles, ensuring ease of application and stable adhesion to the skin surface. The rheological behavior of the two hydrogels reflected a pseudoplastic nature, facilitating smooth, drip-free application. The viscosity recovery after removal of shear stress revealed structural integrity, essential for maintaining product stability during storage and use. These characteristics highlight the suitability of hydrogels for dermatocosmetic applications by combining stability, ease of use, and efficient delivery of the therapeutic benefits of *Lilium* spp. macerates. Both formulations have desirable spreadability and stability, making them highly suitable for dermatocosmetic use. Their rheological performance supports the efficient delivery of the antioxidant and soothing properties of *Lilium* spp. macerate, aligning with user expectations for high-quality skin care products.

Shear stress plotted against shear rate. The curve demonstrates pseudoplastic (shear-thinning) behavior typical for dermatocosmetic gels.

Flow curve for hydrogel HLA-70 depicts non-Newtonian flow characteristics with decreasing viscosity at higher shear rates, confirming pseudoplasticity.

### 2.9. Antioxidant Activity of Hydrogels HLD-70 and HLA-70

This research highlights *Lilium* spp. as a versatile, plant-based ingredient for dermatocosmetic products focused on skin health and resilience (Table 11).

The results confirm that the antioxidant activity of the hydrogels is directly influenced by the phytochemical composition of the macerates used. The better result obtained for HLD-70 is consistent with the superior results obtained for the 70% hydroalcoholic macerate of *Lilium* “Dark Secret”.

## 3. Discussion

In an era dominated by the search for eco-friendly solutions and healthy lifestyles, the use of plant extracts in new dermatocosmetic formulations is gaining more and more ground. This trend is not just a fashion fad, but is based on the proven benefits of natural ingredients in skin care. To date, phytochemical screening of *Lilium* spp. bulb macerates has been less studied concerning their inclusion in various dermatocosmetic formulations. *Lilium candidum*, often referred to as the “Madonna Lily”, has been used to address various medicinal issues, including external treatments for burns and swelling, due to a high content of saponins and polysaccharides that have soothing, anti-inflammatory, and protective properties [4,25]. In traditional medicine, bulbs have been used as poultices to treat ulcers, various wounds, and burned skin [7]; however, some research suggests that the saponins found in *Lilium* spp. (especially from flowers) may actually promote serious skin diseases, though this hypothesis has not been confirmed. On the other hand, Al-bayati et al. (2018) revealed that pyrroline and pyrrolidine alkaloids from *Lilium candidum* can induce oxidative stress and DNA damage, leading to cell apoptosis or necrosis [26]. Additionally, *Lilium candidum* is recognized for its potential in treating cancer and inflammation [4,7]. With a rich history as a medicinal plant, *Lilium candidum* has been the focus of several studies in terms of phytochemical profile, which have led to the isolation and characterization of several new natural products. Therefore, Mimaki et al. (1999) reported, for the first time, the isolation and characterizations of a few natural compounds from bulbs of *Lilium candidum*, such as steroidal saponines, in terms of inhibitory activity on Na+/K+ ATPase. This study revealed that screening compounds for leukemia cell differentiation is linked to Na+/K+ ATPase inhibition [25]. One year later, Eisenreichova et al. (2000) obtained a new alcoholic extract from fresh bulbs of *Lilium candidum* that was concentrated and successively chromatographed over silica gel to obtain a purified new steroidal glycoside [27].

This study presents a comprehensive phytochemical and biological evaluation of *Lilium* spp. bulb macerates, with a particular focus on their potential application in dermatocosmetic formulations, based on their notable antioxidant and antimicrobial properties previously reported in the literature for wild species, but not yet systematically investigated in cultivated varieties. The observed variation in extract performance—dependent on both cultivar and solvent system—offers valuable insights into the phytochemical diversity of *Lilium* spp.

The total phenolic content (TPC) of the analyzed *Lilium* spp. bulb macerates ranged from 30.0 to 225.0 mg GAE/100 g fresh weight (f.w.), reflecting substantial variability among cultivars. These differences suggest a strong genetic influence on the biosynthesis and accumulation of phenolic compounds within the bulbs. When compared to values reported for wild *Lilium* species—typically between 110 and 160 mg GAE/100 g f.w.—several of the cultivars examined in this study demonstrated comparable or even superior phenolic profiles [2]. For instance, *Lilium asiaticum* “White” (LA-70) showed a TPC of 162.5 mg GAE/100 g f.w., consistent with the upper range of literature data [1,2]. The most remarkable result was observed in *Lilium* “Dark Secret” (LD-70), which exhibited a TPC of 225.0 mg GAE/100 g f.w., exceeding previously reported levels for both wild and cultivated *Lilium* species.

The antioxidant activity (AA) of *Lilium* spp. bulb macerates ranged from 3.25 to 81.5 mg GAE/100 g fresh weight (f.w.), reflecting notable interspecies variability. These values correlate with the elevated phenolic and flavonoid contents in these cultivars, confirming their contribution to antioxidant potential. In contrast, the lowest AA was observed in *Lilium candidum* macerated with 96% ethanol (LM-96), which yielded only 3.25 mg GAE/100 g f.w., suggesting a limited antioxidant potential in this specific extract or a low solubility of active components under these extraction conditions.

When compared to literature data, antioxidant activity (AA) in wild *Lilium* species, such as *L. regale*, *L. henryi*, or *L. pumilum*, has typically been reported in the range of 40–70 mg GAE/100 g f.w., depending on species and assay type. The AA value for LD-70, at 81.5 mg GAE/100 g f.w., exceeds this range, indicating superior radical-scavenging potential. These results highlight *Lilium* “Dark Secret” as a particularly promising cultivar, not previously characterized in antioxidant studies, thereby offering new perspectives for its use in formulations targeting oxidative stress and skin protection in dermatocosmetic products.

The flavonoid content (TFC) of *Lilium* spp. bulb macerates revealed significant interspecies variation, underscoring the influence of genetic and metabolic differences on phytochemical accumulation. Among the analyzed cultivars, *Lilium* “Dark Secret” (LD-70) and *L. asiaticum* “White” (LA-70) demonstrated the highest TFC values—26.12 and 21.75 mg QE/100 g f.w., respectively—suggesting their strong potential as sources of antioxidant agents for pharmaceutical and nutraceutical applications. These results align with the literature on wild species such as *L. regale* and *L. pumilum*, which typically present flavonoid contents in the range of 15–22 mg QE/100 g f.w., depending on extraction method and the maturity stage [2]. The slightly higher values observed in LD-70 and LA-70 not only support their robust antioxidant profiles but also highlight the untapped phytochemical potential of ornamental cultivars.

The consistently low TFC values observed in LG-96 and related samples (e.g., 4.37 mg QE/100 g f.w.) may be attributed to a limited intrinsic flavonoid biosynthetic potential or suboptimal extractability of these compounds in the respective genotypes. This pronounced variation among cultivars highlights the critical importance of genotype selection in maximizing the phytochemical yield and functional efficacy of botanical extracts.

Our findings highlight a consistent and statistically significant association between TPC, TFC, and AA, suggesting that these parameters act in concert to enhance the antioxidant efficacy of *Lilium* bulb macerates. This relationship highlights the prominent role of flavonoids as major contributors to the polyphenol content, especially in LD-70, the variety with the highest antioxidant capacity. The abundance of these bioactive compounds may explain the efficacy of the extract in counteracting oxidative processes, directly involved in the onset of skin diseases and aging phenomena. These attributes make *Lilium*-derived macerates valuable candidates for the development of plant-based dermocosmetic formulations [4].

Condensed tannins (CTC) were present in small amounts. Only LD-70 and LM-70 had detectable levels. In other samples, the values were below detection limits. These results differ from what has been reported for *Lilium* flowers. In flowers like those of *Lilium philadelphicum*, tannin and flavonoid levels are much higher. These substances help the plant defend itself against microbes [4].

This difference is linked to the role of the plant organ. Flowers produce more polyphenols for reproduction, UV protection, and defense. Bulbs are storage organs. They accumulate sugars, amino acids, and some phenolic compounds. These usually have simpler structures and fewer tannins. That explains the lower tannin content in bulbs.

The mineral content of the macerates, particularly high levels of potassium, calcium, and magnesium, supports their role in maintaining epidermal hydration, promoting barrier repair, and mitigating inflammation. The absence of toxic heavy metals such as lead and cadmium enhances their safety profile, aligning with regulatory standards for cosmetic ingredients.

FTIR analysis supported the presence of phenylpropanoid derivatives, caffeic acid-related compounds, and hydroxyl-rich structures characteristic of flavonoids and phenolic acids. These findings align with the observed antioxidant activity and offer preliminary evidence of the functional groups responsible for radical scavenging effects.

The antimicrobial activity of *Lilium* spp. bulb macerates appears to be closely linked to their phytochemical composition, particularly the presence of phenolic and flavonoid compounds. These bioactive constituents are known to disrupt microbial cell membranes and interfere with essential metabolic pathways, thereby inhibiting the growth of various pathogens. The antimicrobial evaluation of *Lilium* spp. bulb macerates (LA-70, LD-70, LG-70, LM-70, and LR-70) demonstrated notable efficacy against a range of clinically relevant pathogens, including *Staphylococcus aureus*, *Escherichia coli*, *Pseudomonas aeruginosa*, and *Candida albicans*. The observed efficacy against specific micro-organisms suggests that the antimicrobial properties of these macerates are influenced by both the type and concentration of these compounds. This aligns with the existing literature, which highlights the role of phenolic and flavonoid compounds in the antimicrobial activity of *Lilium* species [1,4,22]. One of the research projects on *Lilium philadelphicum* flowers has demonstrated broad-spectrum antimicrobial activity, suggesting the presence of bioactive compounds effective against various pathogens [22].

Statistical analysis further validated these observations. Significant differences (*p* < 0.05) in TPC, TFC, and AA were confirmed across samples, regardless of solvent, while antimicrobial activity did not show statistically significant differences by sample in single-variable analyses. Two-way ANOVA, however, revealed that antimicrobial efficacy was significantly affected both by the antioxidant activity of the extracts and by the microbial strain, with a notable interaction between these factors (*p* = 0.014). This suggests that, while general antimicrobial trends may be subtle across cultivars, specific combinations of extract bioactivity and microbial susceptibility may yield significant outcomes—an area warranting deeper exploration.

Multivariate statistical approaches provided additional insight. Principal Component Analysis (PCA) indicated that TPC, TFC, and AA strongly co-varied, loading positively onto a single component that explained over 80% of the total variance. In contrast, antimicrobial activity formed a separate dimension, reinforcing the complexity of predicting microbiological outcomes based solely on polyphenol levels. Neural network modeling suggested that TFC and AA were more predictive of antimicrobial performance than TPC or condensed tannins (CTC), pointing to the importance of compound subclass and structure in microbial inhibition.

From a formulation standpoint, hydrogels incorporating LD-70 and LA-70 extracts displayed favorable pH, viscosity, and stability profiles over 60 days. Rheological testing confirmed pseudoplastic behavior, desirable for ease of application, while antioxidant tests on the hydrogels maintained the rank order observed in raw extracts. This suggests that the biological activity is preserved during formulation, and that *Lilium*-based gels could serve as multifunctional topical systems, combining antioxidative protection with favorable application properties [23,24].

The higher antioxidant activity observed in HLD-70 is attributable to its greater phenolic compounds content, which is consistent with previous findings that link phenolic compounds to enhanced free radical-scavenging abilities [16]. This suggests that the *Lilium* “Dark Secret” cultivar may offer superior protective effects against oxidative stress when used in skincare formulations.

Together, these findings highlight the potential of cultivated *Lilium* spp.—notably *L.* “Dark Secret” (LD-70) and *L. asiaticum* “White” (LA-70)—as novel sources of bioactive compounds for use in dermatocosmetic products. This study offers new insight into their antioxidant, antimicrobial, and physicochemical behavior in formulation, while underscoring the importance of species selection, solvent optimization, and extract standardization in natural product development. Future research should focus on the mechanisms underlying the selective antimicrobial effects and evaluate skin permeation and efficacy in in vivo models to further validate the dermocosmetic applications of these plant-based formulations.

Given the results obtained, we propose a future study dedicated to characterizing the phytochemical profile of *Lilium* bulb macerates by integrating HPLC or LC-MS techniques.

## 4. Materials and Methods

The analytical methods selected (Folin–Ciocâlteu assay for phenolics, DPPH assay for antioxidant activity, FTIR spectroscopy for chemical profiling, and agar well diffusion for antimicrobial assays) are widely validated techniques providing reliable and robust quantification of bioactive compounds and their functional activities.

### 4.1. Reagents

All reagents used for chemical determinations were of analytical reagent grade and used without additional purification in the determinations. Gallic acid was purchased from Fluka (Buchs, Switzerland), while the Folin–Ciocâlteu reagent was obtained from Merck (Darmstadt, Germany).

A standard gallic acid solution (34 µg/mL) was prepared, and the Folin–Ciocâlteu reagent was diluted in distilled water at a 1:2 (*v*/*v*) ratio. DPPH (2,2-diphenyl-1-picrylhydrazyl) from Sigma Aldrich (Merck) was prepared as a 0.0063% (1.268 mM) standard solution in methanol, while quercetin from Sigma Aldrich (Merck) was dissolved in methanol as a standard solution (10 mg/100 mL).

Additional reagents used in the study included absolute ethanol (C_2_H_5_OH p.a., min. 99% (*v*/*v*), M = 46.07 g/mol) from Stireco LTH, Buzau, Romania; hydrochloric acid (HCl 30%, based on HCl 37%, ACS.ISO.Reag.Ph.Eur, 1.19 kg/L) from Merck GGaA, Germany; methyl alcohol (CH_3_OH 60%, based on absolute methyl alcohol CH_3_OH, HPLC grade, 0.79 kg/L, M = 32.04 g/mol) from Biosolve Chimie SARL, Dieuze, France; 4% vanillin solution in methanol 60% (*w*/*v*); vanillin (C_8_H_8_O_3_, M = 152.14 g/mol) from Merck GGaA, Germany; and (+)-catechin (C_15_H_14_O_6_, M = 290.28 g/mol) from Carl Roth, Karlsruhe, Germany.

### 4.2. Sample Collection and Preparation

Five different *Lilium* spp. varieties were used in this study, with fresh bulbs purchased from local markets in June 2024, the Dobrogea Region, Romania, near Constanța (44°10′ N, 28°38′ E). The plant material was authenticated by a botanist from the Faculty of Natural Science at the Ovidius University of Constanta, and voucher specimens (Voucher No. RO33-MK, RO-34-MK, RO-35-MK, RO-36-MK, and RO-37-MK) were deposited in the university’s herbarium.

The fresh bulbs were processed for maceration by first being cleaned, dried, and finely chopped. Each sample (10 g) was extracted in 100 mL of ethanol at either 96% (solvent 1) or 70% (solvent 2) concentration and left in contact for 20 days under sealed, dark conditions with daily agitation. After maceration, the samples were filtered, and the resulting macerate solutions were stored at 4 °C in amber glass bottles until further analysis. The filtrates were collected, and their volumes recorded. To standardize the extracted solutions for analysis, all samples were adjusted to a final volume of 100 mL using their respective solvents.

The acronyms for sample identification and solvents are LR-96—*Lilium robina*—ethanol 96%, LG-96—*Lilium* “Sunset boulevard”—ethanol 96%, LA-96—*Lilium asiaticum* “White”—ethanol 96%, LM-96—*Lilium candidum* L. (Madonna Lily)—ethanol 96%, LD-96—*Lilium* “Dark Secret”—ethanol 96%, LR-70—*Lilium robina*—ethanol 70%, LG-70—*Lilium* “Sunset boulevard”—ethanol 70%, LA-70—*Lilium asiaticum* “White”—ethanol 70%, LM-70—*Lilium candidum* L. (Madonna Lily)—ethanol 70%, LD-70—*Lilium* “Dark Secret”—ethanol 70%.

### 4.3. Total Polyphenol Content Analysis

The total polyphenol content of *Lilium* spp. macerates was determined using the Folin–Ciocâlteu method. Absorbance was measured at 681 nm with a UV-VIS Jasco V550 spectrophotometer (JASCO, Tokyo, Japan).

Each 1 mL macerate sample was mixed with 1 mL Folin–Ciocâlteu reagent and 1 mL of 20% sodium carbonate in a 50 mL volumetric flask. After 30 min of incubation, absorbance was recorded.

A calibration curve was constructed using gallic acid standards (0.68–4.72 mg GAE/L), yielding the equation y = 0.1011x − 0.0385 (R^2^ = 0.99909). Results were expressed as mg gallic acid equivalents (GAE) per 100 g fresh weight (f.w.). All measurements were performed in triplicate [28,29,30,31].

### 4.4. Antioxidant Activity Analysis

Antioxidant activity was assessed via the DPPH radical-scavenging assay. For each determination, 1 mL of diluted macerate was combined with 0.1 mL methanol and 3 mL of 0.063% DPPH solution in a 25 mL volumetric flask. The volume was adjusted with methanol, and the mixture was incubated in the dark for 45 min. Absorbance was measured at 530 nm using the same spectrophotometer, with methanol as blank.

The gallic acid calibration curve ranged from 0 to 4.76 mg GAE/L, with the equation y = 1.8976x − 0.3739 and R^2^ = 0.99554. Results were reported as mg GAE/100 g f.w. All samples were analyzed in triplicate [28,29,30,31].

### 4.5. Total Flavonoid Content Analysis

Total flavonoid content (TFC) was assessed using the aluminum chloride colorimetric method. Each 1 mL sample was mixed with 1 mL of 5% NaNO_2_ solution, followed by 1.5 mL of 10% AlCl_3_ after 5 min and 2 mL of 1 M NaOH after an additional 6 min. The volume was adjusted to 25 mL with distilled water, and absorbance was recorded at 510 nm using the UV-VIS spectrophotometer JASCO V550.

Quercetin standards (1.32–7.92 mg QE/L) were used to generate a calibration curve with the equation y = 1.26x + 0.150 (R^2^ = 0.99912). TFC values were expressed as mg quercetin equivalents (QE) per 100 g f.w. All measurements were performed in triplicate [23,24,25,26].

### 4.6. The Condensed Tannins Content (CTC) 

The condensed tannins content was measured in triplicate using the modified vanillin assay proposed by Broadhurst and Jones [15,16] and described in previously published research [20,32]. Appropriate dilutions were made using the extraction solvent (ethanol 70%). The amount of condensed tannins was expressed as mg (+)-catechin equivalents per 100 g of fresh weight (mg CE/100 g f.w.). The calibration curve was made using (+)-catechin as standard (calibration range: 0.015 to 0.500 mg CE/mL; y = 0.572x, correlation coefficient, R^2^: 0.9961). The absorbance was measured at 500 nm using an Evolution 260 Bio UV-Visible spectrophotometer (Thermo Fisher Scientific Inc., Madison, WI, USA); the sample’s absorbance was quantified considering the contributions of the blank and the interference correction sample. The results were given as mean ± standard deviation (SD).

### 4.7. Metal Concentrations Analysis

Metal concentrations in *Lilium* samples were measured using a ContrAA^®^ 700 atomic absorption spectrometer (Analytik Jena GmbH + Co. KG, Jena, Germany), equipped with flame atomization [30]. The *Lilium* spp. bulbs samples were first dried and ground into a fine powder to ensure homogeneity. Approximately 0.5 g of the powdered sample was accurately weighed and subjected to acid digestion using a mixture of 10 mL concentrated nitric acid (HNO_3_) and 2 mL hydrogen peroxide (H_2_O_2_) in a microwave digestion system. This digestion process was carried out to completely break down the organic matrix and release the metal ions into solution. After digestion, the resulting solution was diluted with deionized water to a final volume of 50 mL, ensuring compatibility with the spectrometer.

The mineral content was calibrated using a Certipure^®^ multielement standard solution (Merck, Darmstadt, Germany), containing magnesium, sodium, potassium, calcium, zinc, iron, copper, manganese, nickel, lead, cadmium, and chromium.

To ensure data accuracy and reliability, key performance parameters, including concentration range (µg/L) and calibration curve correlation coefficients (R^2^), were thoroughly evaluated. (Table 12).

### 4.8. FTIR Spectroscopy Analysis

FTIR spectroscopy aims to measure infrared radiation (IR) absorbed by the sample, allowing the investigation of the molecular composition of organic/inorganic compounds. It is well known that an IR spectrum is a fingerprint of a sample in which the absorption peaks are assigned to the different frequencies of vibration (i.e., characteristic modes of vibration presented by molecules) established between the bonds of the atoms of the substances. Generally, each compound represents a combination of atoms/functional groups that have a unique fingerprint and, in consequence, a unique spectrum. This spectral signature is obtained after processing the raw data by Fourier transformation and is presented as a spectrum in a certain interval. The wavelength of each IR vibrational absorbance peak is representative of the intrinsic chemical bonds of the analyzed molecule, making FTIR analysis an excellent tool for chemical characterization. Apart from qualitative investigation, FTIR can quantify the concentration of functional groups from the sample by comparing the intensities of the vibrational bands measured on a sample with calibrated curves. Attenuated Total Reflection-Fourier Transform Infrared spectroscopy (ATR-FTIR) is a vibrational, non-destructive, and non-invasive analytical technique that can be performed in transmission or reflection light mode. Equipped with a diamond ATR (Attenuated Total Reflection) crystal accessory and a Hyperion 3000 microscope, the Vertex 80v FTIR spectrometer (Bruker, Germany) performs analysis of macerate samples in a spectral range of 400–8000 cm^−1^, 0.2 cm^−1^ spectral resolution, and ±1 µm accuracy. An air background spectrum was recorded using 12 co-added scans, with sample spectra subsequently recorded with 64 co-added scans. Signals are collected with a high spatial resolution of a few micrometers. FTIR imaging spectra were baseline corrected, processed, and analyzed using Bruker OPUS v.7.5 software spectra library (Bruker, Germany), under 19% relative humidity [20].

### 4.9. Antimicrobial Assay

To evaluate the antimicrobial efficacy of *Lilium* spp. macerates, a comprehensive in vitro analysis was conducted utilizing the agar well diffusion method, a standard assay for assessing the antimicrobial activity of natural products.

The antimicrobial properties of the macerates were tested against the following reference strains: *Staphylococcus aureus* ATCC 25923 (Gram-positive cocci), *Escherichia coli* ATCC 25922 (Gram-negative bacilli), *Pseudomonas aeruginosa* ATCC 27853 (Gram-negative bacilli), *Candida albicans* ATCC 10231 (yeast). These strains were sourced from the Microorganisms Collection of the Department of Microbiology, Faculty of Biology & Research Institute of the University of Bucharest. Each strain was cultured on appropriate agar media and incubated at 35 ± 2 °C for 18 ± 2 h to ensure optimal growth conditions [33].

A standardized inoculum was prepared by adjusting the turbidity of each microbial suspension to match the 0.5 McFarland standard, corresponding to approximately 1.5 × 10^8^ CFU/mL. This standardization ensures uniformity in microbial load across all assays.

Mueller–Hinton Agar (MHA) plates were prepared following standard protocols, ensuring a uniform depth of 4 mm to facilitate consistent diffusion of the macerates.

The surface of each MHA plate was uniformly inoculated by swabbing with the standardized microbial suspension, ensuring a confluent lawn of growth.

Sterile cork borers were employed to aseptically create wells of 6 mm diameter in the agar.

Volumes of 5 µL, 10 µL, 15 µL, 20 µL, 25 µL, and 30 µL of each *Lilium* spp. macerates (LA-70, LD-70, LG-70, LM-70, LR-70) were carefully dispensed into the respective wells. An alcohol control was included by adding the corresponding solvent volume into a separate well to account for any antimicrobial effects of the solvent.

The inoculated plates were incubated at 35 ± 2 °C for 18 ± 2 h under aerobic conditions to allow for adequate diffusion and interaction between the macerates and the microbial strains.

Post-incubation, the plates were examined for zones of inhibition surrounding the wells. The diameter of each inhibition zone was meticulously measured in millimeters using a calibrated digital caliper to ensure precision. The presence of a clear zone indicated antimicrobial activity, while the absence of such a zone suggested resistance.

The results were systematically recorded by noting the diameter of inhibition zones for each volume of macerate applied. A larger zone of inhibition corresponded to higher antimicrobial activity. The effectiveness of each macerate against the tested micro-organisms was assessed based on these measurements, providing insights into their potential therapeutic applications.

### 4.10. Formulation of Dermatocosmetic Gels with Lilium spp. Bulb macerates

#### Pre-Formulation Conditions

*Lilium* spp. bulbs, species LD-70—*Lilium* “Dark Secret”—ethanol 70% and LA-70—*Lilium asiaticum* “White”—ethanol 70%, were chosen for obtaining dermatocosmetic gels due to their superior antioxidant activity and high total polyphenol content.

Different concentrations of the two species of *Lilium* spp. bulbs macerate (e.g., 0.5%, 1.0%, 2.0%, 2.5%, and 5.0% *w*/*w*) were tested by direct incorporation into the gel. Based on the experimental results, 5% *w*/*w Lilium* spp. bulbs macerate was identified as optimal for gels prepared with 2.0% Carbopol 940. This concentration provided sufficient antioxidant activity for protective effects, balanced antimicrobial efficacy without compromising skin compatibility, good viscosity, and gradability. The chosen concentrations ensured that the formulations were both effective and consumer-friendly.

Preparation: Two hydrogel formulations (HLD-70 and HLA-70) were prepared with the composition shown in Table 13. Carbopol 940, corresponding to a final concentration of 2.0%, was hydrated using purified water for at least 24 h in the presence of glycerin as a dispersant (purity > 99%; Glycerin from Merck, Darmstadt, Germany). Triethanolamine (purity > 99%; Triethanolamine from Carl Roth GmbH + Co. KG., Karlsruhe, Germany) was used for neutralization at the end of 24 h in the cold and was added to the semisolid matrix generated by hydration of the hydrophilic polymer under intense stirring (2000 rpm for 10 min, in a Hedolph R20R20R20R turbine stirrer) (Heidolph Instruments GmbH & Co. KG, Schwabach, Germany). Finally, *Lilium* spp. bulbs macerate was added to the semisolid matrix under stirring until complete homogenization.

Hydrogels with the hydroalcoholic macerates of the two species of *Lilium* spp. bulbs were prepared by dispersing 2 g of Carbopol 940 in distilled water and glycerin. Each preparation was mixed vigorously and then allowed to hydrate for 30–60 min. Subsequently, a 5% hydroalcoholic macerate of *Lilium* spp. bulbs species was added to the Carbopol suspension, and continuously mixed for uniform distribution. When the mixture became homogeneous, the pH of the gel was adjusted to a value of 5.0–5.5 using a triethanolamine (TEA) solution. After preparation, the gel was kept in a refrigerator for 24 h to allow stabilization and ensure optimal development of its properties.

### 4.11. Characterization of Gel Preparations

Characterization of the obtained hydrogels involved several analyses, including visual inspection, pH measurement, rheological behavior, and spreadability evaluation [23].

The formulations were mixed well before measurement, ensuring a uniform pH distribution in the sample. The pH was recorded after a 30 s stabilization period, and three readings were taken for each formulation. If the pH readings deviated by more than ±0.1 pH unit, the sample was remeasured.

Appearance analysis: Appearance was assessed by spreading a thin layer of the sample on a microscope slide and examining it under a magnifying glass (4.5× magnification) to observe texture and uniformity.

For pH measurement, samples were extracted in a ratio of 1:10 with distilled water, heated in a water bath at 60 °C, and homogenized for 10 min. The aqueous phase was then separated, and the pH was recorded using a multiparameter pH meter from Hanna Instruments (Hanna Instruments, Woonsocket, RI, USA).

Spreading capacity assessment: The degree of spreading was assessed 30 h after formulation. For this determination, 1 g hydrogel was placed between two 20 × 20 cm glass plates for 1 min, following the Ojeda Arbussa method [23]. A standardized 125 g top plate was initially used, and additional weights (50 g, 100 g, 150 g, 200 g, 250 g) were gradually applied at 1 min intervals. The spread diameters were measured in millimeters. The experiment was repeated 30 and 60 days after obtaining the hydrogels. The results were expressed as spread area in relation to the applied weight, calculated using Equation (1).

Si = di 2 (π/4)(1)

where:

- Si is the scattering area (mm^2^) resulting from the applied mass “i” (g), and
- di is the average diameter (mm) reached by the sample.

### 4.12. Rheological Measurements

The hydrogels were subjected to a detailed rheological analysis using a cylindrical system with a defined geometry. Using the mathematical models presented in Equations (2)–(6), key rheological parameters were calculated. The apparent viscosity was determined by Equation (2), allowing for the creation of viscosity curves. Rheograms for the dermatocosmetic formulations were generated using Equation (3), which related the shear stress (τ) to the shear rate (D). The shear rate (D, in s^−1^) was calculated by Equation (4) based on the rotation speed (ω, in rpm). A specific constant (R) was applied for each axis of rotation to derive D from ω. In addition, the shear stress (τ) was determined using Equation (5).

The rheological properties were evaluated at various rotational speeds (ω) between 4 and 200 rpm. The measurements were performed with a rotational viscometer ST-2020 R, manufactured by Laboquimia, Barcelona, Spain, using 10 s intervals for each determination. Viscosity evaluations were performed with spindles R5 and R6, which were chosen to match the viscosity range of the samples. These axes facilitated the calculation of the shear rate (D) in relation to the rotational speed (ω). Concerning the comparison of the results with a known rheological model (Ostwald de Waele), Equation (6) was applied [23].

η = f(D)(2)

D = f(τ)(3)

D = ω × R(4)

τ = η × D(5)

τ = k × D^n^ (6)

### 4.13. Determination of Antioxidant Activity

The antioxidant activity of hydrogel formulas (HLD-70 and HLA-70) was evaluated using the DPPH radical-scavenging assay, with gallic acid (GAE) as the standard for plotting calibration curves [28]. The results obtained for the two formulated hydrogels (μg GAE/100 g gel) are presented in Table 11.

The calibration curve for gallic acid was linear across the concentration range of 0–4.08 mg GAE/L, with a correlation coefficient (R^2^) of 0.9983, calibration curve equation: y = 2.368 − 0.0981x.

For the antioxidant activity assay, 1 mL hydrogel diluted with 0.1 mL ethanol was mixed with 3 mL 0.063% DPPH solution in a 25 mL volumetric flask. The solution was brought to volume with methanol and incubated in the dark for 45 min. Absorbance was then measured at 530 nm.

### 4.14. Statistical Analysis

Factor Analysis, Principal Component Analysis (PCA) and Neural Network Analysis were employed to interpret the complex relationships among phytochemical content, antioxidant properties, and antimicrobial effects due to their effectiveness in managing multidimensional datasets and non-linear correlations.

Statistical analysis was conducted using IBM SPSS Statistics v. 26. *t*-test, One-Way ANOVA using the Duncan post-hoc test, Two-Factor ANOVA (with a significance threshold *p* < 0.05), Pearson’s correlation analysis, Factor Analysis, Hierarchical Cluster Analysis, and Neural Network Analysis were performed. The statistical analysis was applied both for the phytochemical and microbiological parameters characterizing the *Lilium* spp. macerates.

## 5. Conclusions

The comprehensive evaluation conducted in this study highlights the significant phytochemical diversity, robust antioxidant activity, favorable mineral safety profiles, characteristic spectroscopic attributes, and notable antimicrobial activities of *Lilium* spp. bulb macerates. Notably, moderate ethanol concentration (70%) was determined to be more effective in extracting bioactive compounds compared to a higher ethanol concentration (96%). Among the tested varieties, macerates derived from *Lilium* “Dark secret” (LD-70) and *Lilium asiaticum* “White” (LA-70) demonstrated superior antioxidant and antimicrobial capacities, positioning these cultivars as promising candidates for natural therapeutic and cosmetic formulations.

The study also confirms the stability and optimal physicochemical properties of dermatocosmetic hydrogels formulated with LD-70 and LA-70 macerates, ensuring their suitability for long-term dermal application.

Furthermore, the optimization of extraction protocols and clinical evaluations are recommended to facilitate the effective translation of these promising findings into practical pharmaceutical, nutraceutical, and cosmetic applications.

## Figures and Tables

**Table 1:** Condensed tannins content (CTC) for macerates of *Lilium* spp. bulbs.

Sample Code	CTC * [mg CE/100 g f.w.]
LR-70	n.a.
LG-70	n.a.
LA-70	n.a.
LM-70	34.97 ± 17.48
LD-70	46.62 ± 10.09

* mean ± standard deviation (triplicate); n.a.—not detectable.

**Table 2:** Pearson correlation matrix between antioxidant activity (AA), total polyphenolic content (TPC), and total flavonoid content (TFC) in *Lilium* spp. bulb macerates.

Variables	TPC	AA	TFC
TPC	1	0.907 *	0.839 *
AA	0.907 *	1	0.895 *
TFC	0.839 *	0.895 *	1

* Correlation is significant at the 0.01 level (2-tailed).

**Table 3:** Relative intensity and tentative assignments of *Lilium* spp. macerates (i.e., *Lilium robina* (LR), *Lilium* “Dark secret” (LD), *Lilium* “Sunset boulevard” (LG), *Lilium candidum* L. (Madonna Lily) (LM), and *Lilium asiaticum* “White” (LA)).

Wavenumber [cm^−1^]/Relative Intensity *					Tentative Assignments
LR	LD	LG	LM	LA	
878m	879m	878m	879m	878m	C-H bending of substituted aromatic ring
949w	952w	950w	952w	950w	C-H bending of substituted aromatic ring
1044s	1045s	1044s	1045s	1044s	O-H of carboxyl group
1086m	1087m	1086m	1087m	1086m	C-O stretching of single bond of alcohol
1128w	1129w	1128w	1129w	1128w	C-C stretching of substituted ring
1159w	1160w	1160w	1160w	1160w	C-O stretching vibration combined with aromatic ring
1274w	1274w	1274w	1274w	1274w	C-C in-phase (symmetric motion) ring stretching
1324w	1325w	1325w	1326w	1324w	C-O stretching vibration combined with aromatic ring
1382w	1380w	1382w	1380w	1382w	bending vibrations of CH_2_
1414w	1416w	1415w	1416w	1415w	bending vibration of the O-H of alcohol
1453w	1453w	1453w	1453w	1453w	bending vibrations of CH_2_
1647w	1652w	1647w	1654w	1647w	C=C stretching vibration of double bond
2897w	2885w	2897w	2884w	2896w	bending vibration of the O-H of phenol
2929w	2927w	2930w	2928w	2929w	C-O/C=O of carbonyl/carboxyl groups
2975m	2973m	2975w	2973m	2975m	bending vibration of the O-H of phenol
3335s	3328m	3342m	3329m	3341s	bending vibration of the O-H of alcohol

* w—weak; m—medium; s—strong.

**Table 4:** Antimicrobial activity of *Lilium* spp. bulb macerates.

Organism	Volume [µL]	Sample—Inhibition Zone [mm]					
		LA-70 Sensitivity (S)/ Resistance (R)	LD-70 Sensitivity (S)/ Resistance (R)	LG-70 Sensitivity (S)/ Resistance (R)	LM-70 Sensitivity (S)/ Resistance (R)	LR-70 Sensitivity (S)/ Resistance (R)	Control Sensitivity (S)/ Resistance (R)
*Staphylococcus aureus* ATCC 25922	5	0 (R)	0 (R)	8 (S)	0 (R)	0 (R)	0 (R)
	10	0 (R)	0 (R)	11 (S)	0 (R)	0 (R)	0 (R)
	15	0 (R)	0 (R)	13 (S)	0 (R)	0 (R)	0 (R)
	20	12 (S)	10 (S)	14 (S)	0 (R)	0 (R)	0 (R)
	25	15 (S)	10 (S)	15 (S)	0 (R)	0 (R)	0 (R)
	30	17 (S)	14 (S)	18 (S)	0 (R)	0 (R)	0 (R)
*Escherichia coli* ATCC 25923	5	9 (S)	9 (S)	8 (S)	8 (S)	8 (S)	8 (S)
	10	12 (S)	11(S)	12(S)	12 (S)	12 (S)	10 (S)
	15	14(S)	13 (S)	13–14(S)	12 (S)	15–16 (S)	12 (S)
	20	16 (S)	15 (S)	15(S)	13 (S)	16 (S)	14 (S)
	25	18 (S)	16 (S)	17(S)	16 (S)	17 (S)	15 (S)
	30	19 (S)	17 (S)	18(S)	17 (S)	18 (S)	16 (S)
*Pseudomonas aeruginosa* ATCC 27853	5	8 (S)	9 (S)	8 (S)	8 (S)	8 (S)	7 (S)
	10	12 (S)	11 (S)	12 (S)	11(S)	12 (S)	9 (S)
	15	15 (S)	13 (S)	14 (S)	13 (S)	13 (S)	10 (S)
	20	16 (S)	15 (S)	16 (S)	16 (S)	15 (S)	14 (S)
	25	19 (S)	17 (S)	18 (S)	16 (S)	16 (S)	15 (S)
	30	21(S)	19(S)	20 (S)	18 (S)	18 (S)	16 (S)
*Candida albicans* ATCC 10231	5	10 (S)	10 (S)	10 (S)	9 (S)	9 (S)	9 (S)
	10	13 (S)	12 (S)	13 (S)	11(S)	11(S)	11(S)
	15	14 (S)	15 (S)	15 (S)	13(S)	13 (S)	13 (S)
	20	17 (S)	16 (S)	18(S)	14(S)	15 (S)	15 (S)
	25	20 (S)	19 (S)	19 (S)	16 (S)	17 (S)	17 (S)
	30	22 (S)	20 (S)	20 (S)	20 (S)	20 (S)	20 (S)

0—No inhibition zone observed.

**Table 5:** Pearson correlation analysis between the chemical parameters and antimicrobial activity of *Lilium* spp. macerates (70% ethanol).

Variables	TPC *	AA *	TFC *	CTC *	Inhibition Zone
TPC *	1	0.861 **	0.876 **	0.404 **	0.056
AA *	0.861 **	1	0.865 **	0.171	−0.011
TFC *	0.876 **	0.865 **	1	0.310 **	0.063
CTC *	0.404 **	0.171	0.310 **	1	−0.110
Inhibition zone	0.056	−0.011	0.063	0.110	1

* TPC—Total polyphenolic content (mg gallic acid/100 g of fresh weight); AA—antioxidant activity (mg gallic acid/100 g fresh weight); TFC—Total flavonoid content (mg quercetin/100 g fresh weight), CTC—Condensed tannins content (mg (+)-catechin eq./100 g of fresh weight). ** Correlation is significant at the 0.01 level (2-tailed).

**Table 6:** Factor loadings (Varimax with Kaiser normalization) using Principal Component Extraction.

Factor	Component 1	Component 2
Eigenvalue	2.875	1.073
Cumulative variance (%)	56.78	78.96
TPC *	0.958	−0.103
AA *	0.925	−0.031
TFC *	0.952	−0.044
CTC *	0.368	−0.585
Inhibition zone	0.153	0.868

* TPC—Total polyphenolic content (mg gallic acid/100 g of fresh weight); AA—antioxidant activity (mg gallic acid/100 g fresh weight); TFC—Total flavonoid content (mg quercetin/100 g fresh weight); CTC—Condensed tannins content (mg (+)-catechin eq./100 g of fresh weight).

**Table 7:** Parameter Estimates (predicted) in the frame of Multilayer Perceptron.

Predictor		Hidden Layer 1				Output Layer (Strengthens of Antimicrobial Activity)		
		H(1:1)	H(1:2)	H(1:3)	H(1:4)	0	1	2
Input Layer	(Bias)	1.762	−2.251	−0.280	2.043			
	TPC	2.062	−0.004	−1.059	0.394			
	AA	−0.758	1.270	0.618	−0.281			
	TFC	0.687	−1.309	−0.145	0.329			
	CTC	0.498	0.623	−0.063	−0.704			
	Volume	−0.626	−0.086	−0.947	4.049			
	Organism	0.561	−2.297	−2.023	0.634			
Hidden Layer 1	(Bias)					−0.940	0.345	0.104
	H(1:1)					−1.633	2.544	−0.764
	H(1:2)					4.430	−1.155	−3.076
	H(1:3)					2.251	0.121	−2.558
	H(1:4)					−2.416	−1.490	3.636

**Table 8:** Characteristics of G based on *Lilium* spp. macerates.

Characteristics	Formula HLD-70 *	Formula HLA-70 *
Organoleptic evaluation—after 24 h	appearance: homogeneous, translucent; color: dark yellow; smell: specific	appearance: homogeneous, translucent; color: yellow-light; smell: specific
Organoleptic evaluation—after 30 days	constant initial characteristics	constant initial characteristics
Organoleptic evaluation—after 60 days	constant initial characteristics	constant initial characteristics
pH—after 24 h	4.6–4.9	4.8–5.0
pH—after 30 days	5.0–5.2	5.1–5.4
pH—after 60 days	5.4	5.6
Viscosity—after 24 h	690 ± 0.88 mPa·s	642 ± 0.88 mPa·s
Viscosity—after 30 days	670 ± 0.25 mPa·s	628 ± 0.22 mPa·s
Viscosity—after 60 days	628 ± 0.76 mPa·s	608 ± 0.46 mPa·s

* HLD-70—hydrogel of *Lilium* “Dark Secret”—ethanol 70% macerate; HLA-70—hydrogel of *Lilium asiaticum* “White”—ethanol 70% macerate.

**Table 9:** Value intervals for rheological parameters.

Sample	Shear Spead D [s^−1^] The Interval	Viscosity ƞ [cP] The Interval	Shear Stress τ [mPa] The Interval
HLD-70 *	6.10–78	2600–34,200	682,500–140,380
HLA-70 *	5.8–62	820–48,812	48,400–146,820

* HLD-70—hydrogel of *Lilium* “Dark Secret”—ethanol 70% macerate; HLA-70—hydrogel of *Lilium asiaticum* “White”—ethanol 70% macerate.

**Table 10:** The coefficient values of the Ostwald de Waele Rheological Model.

Sample	K Consistency Coefficient	n Flow Coefficient	R Correlation Coefficient Ostwald de Waele
HLD-70 *	14,624	0.5221	0.9973
HLA-70 *	12,164	0.4632	0.9982

* HLD-70—hydrogel of *Lilium* “Dark Secret”—ethanol 70% macerate; HLA-70—hydrogel of *Lilium asiaticum* “White”—ethanol 70% macerate.

**Table 11:** Antioxidant activity of hydrogels HLD-70 and HLA-70.

No	Sample	DPPH [μg/100 g Gel]
1.	HLD-70 *	142.4 ± 1.8%
2.	HLA-70 *	86.8 ± 6.3%

* HLD-70—hydrogel *Lilium* “Dark Secret”—70% ethanol; HLA-70—hydrogel of *Lilium asiaticum* “White”—ethanol 70%.

**Table 12:** Performance parameters for AAS measurements.

Metal	Concentration Domain (mg/L)	R^2^	Calibration Curve Equation
Cadmium	0.0004–0.004	0.9989	y = 0.0122973x + 0.0422208
Calcium	40.00–200.00	0.9996	y = 0.000138x + 0.0000701
Chromium	0.002–0.02	0.9913	y = 0.0138310x + 0.0125257
Copper	0.003–0.03	0.9958	y = 0.1937482x + 0.0141176
Iron	0.05–2.0	0.9929	y = 0.0007637x + 0.0308354
Lead	0.01–0.1	0.9976	y = 0.0145906x + 0.0042433
Magnesium	1.0–5.0	0.9932	y = 0.0062880x + 0.0571131
Manganese	0.0015–0.015	0.9950	y = 0.0129916x + 0.0202151
Nickel	0.007–0.07	0.9926	y = 0.0107338 x + 0.0041630
Potassium	1.0–5.0	0.9975	y = 0.0013933x + 0.0024000
Sodium	5.0–25.0	0.9966	y = 0.0000076x + 0.0034889
Zinc	0.0005–0.005	0.9925	y = 0.3079303x + 0.0533993

**Table 13:** Composition of hydrogels with *Lilium* spp. bulbs macerated in 70% ethanol.

Components	Mass [g]	
	HLD-70 *	HLA-70 *
Carbopol 940	2.0	2.0
Glycerine	5	5
Triethanolamine	q.s. **	q.s. **
Hydroalcoholic macerate of *Lilium* spp. bulbs	5.0	5.0
Purified water	until 100	until 100

* HLD-70—hydrogel of *Lilium* “Dark Secret”—ethanol 70% macerate; HLA-70—hydrogel of *Lilium asiaticum* “White”—ethanol 70% macerate; ** q.s. = quantum sufficit.

## Data Availability

The original contributions presented in the study are included in the article; further inquiries can be directed to the corresponding author.
